# Regulatory roles of microRNA-708 and microRNA-31 in proliferation, apoptosis and invasion of colorectal cancer cells

**DOI:** 10.3892/ol.2014.2328

**Published:** 2014-07-09

**Authors:** SAN-LIN LEI, HUA ZHAO, HONG-LIANG YAO, YONG CHEN, ZHEN-DONG LEI, KUI-JIE LIU, QUN YANG

**Affiliations:** 1 Department of General Surgery, The Second Xiangya Hospital of Central South University, Changsha, Hunan 410011, P.R. China; 2 Department of Oncology, The Second Xiangya Hospital of Central South University, Changsha, Hunan 410011, P.R. China

**Keywords:** microRNA-708, microRNA-31, oncogene, colorectal cancer, cyclin-dependent kinase inhibitor 2B

## Abstract

MicroRNAs (miRs) function as key regulators of gene expression and their deregulation is associated with the carcinogenesis of various cancers. In the present study, the aim was to validate the potential roles and regulatory mechanisms of miR-708 and miR-31 in colorectal cancer (CRC) cells. miR-708 and miR-31 were found to be highly expressed in five CRC tissue samples. Functional studies showed that the inhibition of miR-708 and miR-31 inhibited cell proliferation and invasion, however, promoted apoptosis *in vitro*. Subsequently, it was identified that miR-708 and miR-31 directly target cyclin-dependent kinase inhibitor 2B (CDKN2B) by binding to the 3′ untranslated region, which suppresses the CDKN2B protein levels. In addition, the CDKN2B protein levels were significantly reduced when there was high miR-708 and miR-31 expression in the CRC tissue samples. The results indicate that miR-31 and miR-708 function in an oncogenic manner in CRC development, and inhibition of the two miRs may be used as a therapeutic strategy for patients with CRC.

## Introduction

Colorectal cancer (CRC) is one of the most common malignant cancers and is the second leading cause of cancer-related mortality worldwide (1). The five-year survival rate of patients in the early stage is high, while that of late-stage patients is low due to metastasis. The underlying molecular mechanism of metastasis is yet to be elucidated, therefore, it is important to investigate and validate the novel biomarkers that are involved in CRC development. Recently, microRNAs (miRs) have been identified as novel molecules that are crucial in cancer development (2–4).

miRs are a family of endogenous, small (containing ~22 nucleotides) non-coding RNA molecules, which are able to serve as key regulators of gene expression at the post-transcriptional level (5). They are able to bind to the target gene mRNA in a complete or incomplete complementary manner with the 3′ untranslated region (UTR) of the target gene mRNA, leading to target mRNA degradation or translational repression (5,6). Due to the incomplete base pair between the miR and the 3′UTR of the target gene, one gene may be regulated by multiple miRs, resulting in a complex regulation network of miRs. Thus far, ~60% of the protein-coding genes are known to be regulated by miRs and, depending on the roles of the target genes, the miR functions as an oncogene or a tumor suppressor (7). Numerous studies have shown that miRs participate in various biological processes, including cell growth, timing development, apoptosis and differentiation (8–11). Furthermore, studies indicated that the deregulation of miRs is associated with cancer initiation and development, including miR-122 in liver cancer (12,13), miR-365 (14) and miR-25 (15) in colorectal cancer, and miR-125b in breast cancer (16).

In the present study, the aim was to investigate the putative roles of miR-708 and miR-31 in CRC. In addition, 3-(4,5-dimethylthiazol-2-yl)-2,5-diphenyltetrazolium bromide and colony formation assay, as well as other functional assays were used to elucidate the miR-708 and miR-31 in CRC cells. Furthermore, an attempt was made to identify the downstream genes of these two miRs, which are likely to aid the identification of a novel therapeutic strategy for patients with CRC.

## Materials and methods

### Tissue samples and cell culture

Five pairs of human CRC tissue and the adjacent healthy tissue were supplied by The Second Xiangya Hospital of Central South University (Changsha, China). The tissue samples were confirmed by observation of the morphology and immunohistochemistry, and consent was obtained from the patients with CRC. The tissues were stored at −80°C.

The human CRC cell line, SW480, was cultured in Dulbecco’s modified Eagle’s medium supplemented with 10% fetal bovine serum (FBS) and 2 mM L-glutamine (Invitrogen Life Technologies, Carlsbad, CA, USA). The SW620 cells were cultured in L-15 medium (Shanghai Haoran Bio Technologies Co., Ltd., Shanghai, China) supplemented with 10% FBS and all of the cells were maintained at 37°C in a 5% CO_2_ atmosphere.

### Transfection

The anti-sense oligonucleotides of miR-708, miR-31, miR-708 and miR-31 mimics, and the controls [anti-negative control (NC) or NC] were purchased from Genepharma Co., Ltd. (Shanghai, China). The cells were transfected with the abovementioned oligonucleotides by Lipofectamine™ 2000 (Invitrogen Life Technologies) according to the manufacturer’s instructions.

### RNA isolation and real-time polymerase chain reaction (qPCR) analysis

The total RNA (including miR) was isolated using the Trizol reagent (Invitrogen Life Technologies) according to the manufacturer’s instructions. The RNA concentration was determined using a NanoDrop-1000 spectrophotometer (Thermo Scientific, Rockford, IL, USA). For the reverse transcription (RT) reaction of the miR, specific miR RT primers were used. U6 small nuclear B non-coding RNA served as an internal control. qPCR was performed using the SYBR Green PCR master mix (Applied Biosystems, Foster City, CA, USA) according to the following conditions: 95°C for 5 min followed by 40 cycles of amplification at 95°C for 30 sec, 57°C for 30 sec and 72°C for 30 sec.

### Western blot analysis

The cells were plated into 6-well plates (Jixing Biocompany, Shanghai, China) at a density of 30×10^4^ cells/well and transfected on the second day when the cell confluence reached ~80%. At 48 h after transfection, the cells were lysed by radioimmunoprecipitation assay (RIPA) buffer (50 mM Tris-HCl, pH 8.8, 150 mM NaCl, 1% NP-40, 1% sodium deoxycholate and 0.1% SDS) for 30 min at 4°C. The protein concentration was measured using the bicinchoninic acid method. The first antibody was rabbit monoclonal anti-cyclin-dependent kinase inhibitor 2B (CDKN2B) antibody (1:200 dilution; Abcam, Cambridge, MA, USA) and anti-GAPDH antibody (1:1000 dilution; Abcam). The secondary antibody was goat anti-rabbit immunoglobulin G conjugated with horseradish peroxidase (1:1000 dilution). The bound antibodies were detected with the ECL Plus Western Blotting Detection system (GE Healthcare, Princeton, NJ, USA) and the chemiluminescent signals were detected with a high-performance chemiluminescence film (GE Healthcare). GAPDH served as an internal control to normalize the CDKN2B protein levels.

### The 3-(4,5-dimethylthiazol-2-yl)-2,5-diphenyltetrazolium bromide (MTT) assay

The transfected cells were plated into 96-well plates (Jixing Biocompany) at a density of 5×10^3^ cells/well. At 24, 48 and 72 h after transfection, the cells were incubated with MTT reagent (Xinshiye Biocompany, Guangzhou, China) for ~4 h at 37°C. Subsequently, the supernatant was replaced with dimethyl sulphoxide to dissolve solid residues. A spectrophotometer was used to measure the absorbance at 570 nm.

### Apoptosis assay

Camptothecin (Sigma Aldrich, St. Louis, MO, USA) was added to the cell medium following transfection with anti-miR-708 or anti-miR-31. Following incubation for 24 h, the cells were collected and detected using an Annexin V fluorescein isothiocyanate kit on a BD FACSCalibur™ system (Becton-Dickinson, Franklin Lakes, NJ, USA) according to the manufacturer’s instructions.

### DNA constructs and luciferase reporter assay

The 3′UTR of CDKN2B was amplified and inserted downstream of the luciferase reporter gene. The mutant 3′UTR of CDKN2B (various nucleotides within the binding sites were mutated) was amplified using CDKN2B 3′UTR as the template. The cells were co-transfected with miR mimics and CDKN2B 3′UTR or mutant 3′UTR, together with the controls. At 48 h after transfection, the cells were collected and lysed using the RIPA buffer. The luciferase intensity was measured using the Dual Luciferase Reporter assay system (Promega Corporation, Madison, WI, USA) according to the manufacturer’s instructions.

### Transwell invasion assay

The cell invasion ability was performed using the Transwell chamber with Matrigel (Millipore, Billerica, MA, USA). The transfected cells were plated into the upper chamber with 250 μl serum-free medium, while the lower chamber was filled with 750 μl cell medium with 10% FBS. When the cells had been invaded for ~20 h, the cells were wash, fixed and stained with 5% crystal violet (Sigma Aldrich). The cells that had not invaded the membrane were removed using cotton tips. Finally, the invasive cells were imaged (EOS 500D, Canon, Tokyo, Japan) and counted under a microscope (CSW-17AD, Cosway (China) Co., Ltd., Shenzen, China)

### Statistical analysis

The difference between groups was analyzed by Students’ t-test, and P<0.05 was considered to indicate a statistically significant difference. All the data were represented as means ± standard deviation.

## Results

### miR-708 and miR-31 are upregulated in CRC tissues

**Figure 1:**
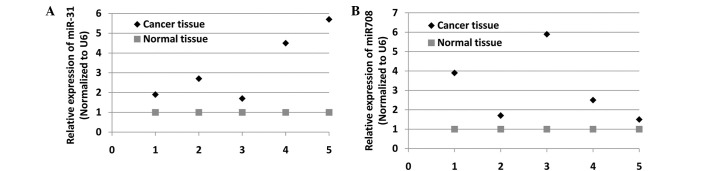
Expression of miR-31 and miR-708 was positively associated with colon cancer. (A) miR-31 and miR-708 are upregulated in all five paired CRC tissues compared with adjacent healthy colon tissues. (B) *In situ* hybridization showing the upregulation of miR-31 and miR-708 in CRC tissues. miR, microRNA; CRC, colorectal cancer; U6, RUN6B.

In order to determine the potential roles of miR-708 and miR-31 in CRC cells, the expression levels of the two miRs were evaluated in five pairs of CRC tissue and the adjacent healthy tissue using qPCR analysis. Fig. 1A and B show that miR-31 and miR-708 were highly expressed in CRC tissues, respectively. Therefore, these data indicate the possible significance of miR-708 and miR-31 in CRC development.

### Inhibition of miR-708 and miR-31 suppresses cell proliferation

**Figure 2:**
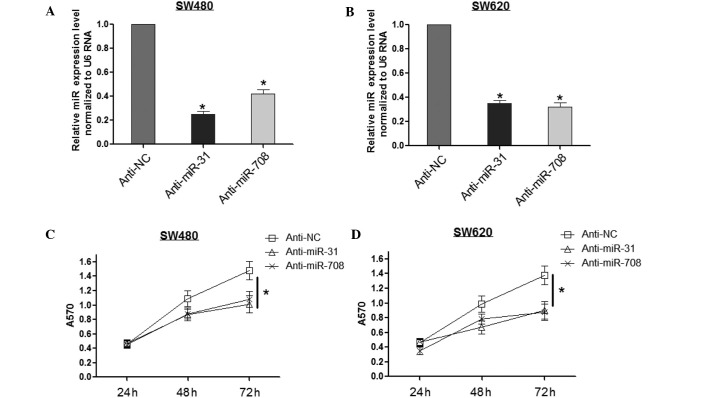
miR-31 and miR-708 regulate cell proliferation in the CRC cell lines, SW480 and SW620. (A and B) Downregulation of miR-31 and miR-708 by transient transfection of anti-miR-31 or anti-miR-708 in the SW480 and SW620 cells. (C and D) The cell proliferation was analyzed using the MTT assay in the two cell lines transfected with anti-miR-31 or anti-miR-708. ^*^P<0.05 vs. NC. miR, microRNA; CRC, colorectal cancer; NC, negative control; A570, absorbance at 570 nm.

The MTT assay was performed to investigate the roles of miR-708 and miR-31 in CRC cell proliferation. First, miR-708 and miR-31 levels were inhibited by anti-miR transfection. Fig. 2A and B show that anti-miR-31 led to the reduction of miR-31 by ~30% in the SW480 and SW620 cells, and anti-miR-708 reduced the miR-708 expression by ~40%. Secondly, the MTT assay was conducted in the SW480 and SW620 cells. As shown in Fig. 2C, anti-miR-31 and anti-miR-708 inhibited the SW480 cell viability at 72 h after transfection, while no effect was observed at 24 and 48 h. Consistently, there were similar effects of miR-708 and miR-31 identified in the SW620 cells (Fig. 2D), indicating that miR-708 and miR-31 may be significant in CRC development.

### Inhibition of miR-708 and miR-31 promotes cell apoptosis

**Figure 3:**
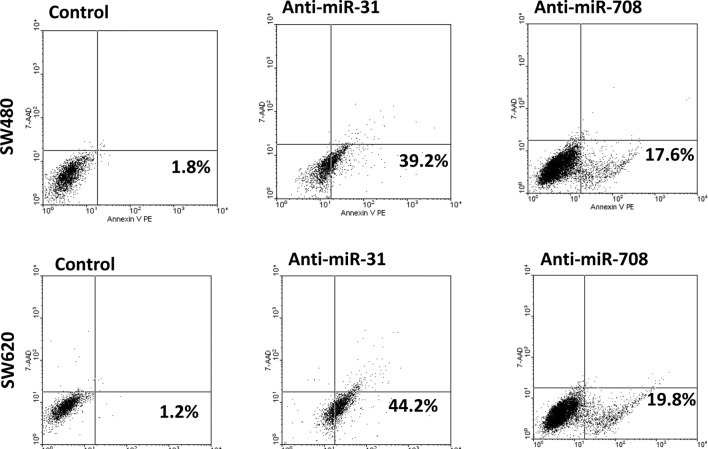
Anti-miR-31 and anti-miR-708 enhance the apoptosis of human cell line cells, SW480 and SW620. The percentage of apoptotic cells was determined by flow cytometry. In the SW480 and SW620 cells, the percentage of apoptotic cells increased to 39.2 and 44.2%, respectively by transient transfection of anti-miR-31, while the percentage of apoptotic cells increased to 17.6% and 19.8%, respectively by transient transfection of anti-miR-708. miR, microRNA.

The effect of miR-708 and miR-31 on cell apoptosis was further investigated using the Annexin V assay. Fig. 3 demonstrates that anti-miR-31 significantly increased the apoptosis of the SW480 and SW620 cells, and anti-miR-708 led to similar results. Overall, these data indicate that miR-708 and miR-31 may serve as significant anti-apoptosis factors in CRC.

### Inhibition of miR-708 and miR-31 suppresses cell invasion

**Figure 4:**
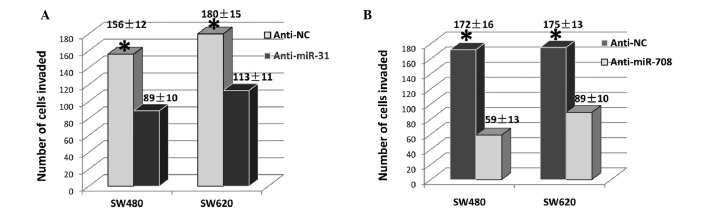
miR-31 and miR-708 regulate cell invasion of CRC cells *in vitro*. (A) Transwell invasion assay of human cell line cells, SW480 and SW620 transfected with anti-NC or anti-miR-31. (B) Transwell invasion assay of SW480 and SW620 cells transfected with anti-NC or anti-miR-708. ^*^P<0.05 vs. anti-miR-NC. miR, microRNA; CRC, colorectal cancer; NC, negative control.

As cell invasion is an important factor for tumor metastasis, the effect of miR-708 and miR-31 on cell invasion was subsequently investigated. As shown in Fig. 4A, anti-miR-31 reduced the number of invasive cells by ~40% in the SW480 and in the SW620 cells, compared with the controls. Similarly, anti-miR-708 reduced the invasive cells by ~80 and ~50%, in the SW480 and SW620 cells, respectively (Fig. 4B). These results indicate that miR-708 and miR-31 may be significant in CRC metastasis.

### miR-708 and miR-31 directly target CDKN2B by binding to the 3′UTR

**Figure 5:**
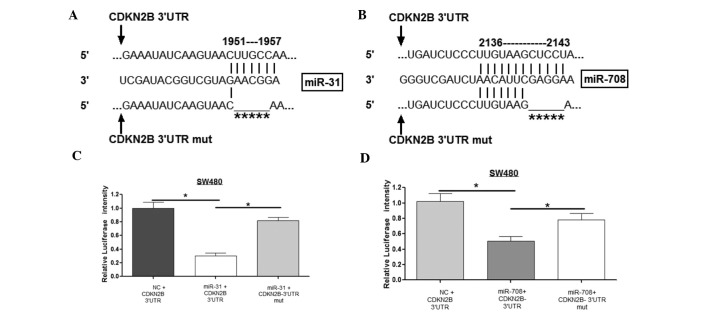
Identification of CDKN2B as a direct target gene for miR-31 and miR-708. (A and B) The 3′UTR of CDKN2B mRNA possesses binding sites for miR-31 or miR-708 and five nucleotides within the binding sites were mutated. (C and D) The SW480 human cell line cells were co-transfected with wild type or mutant CDKN2B 3′UTR and either miR-31 or NC miR mimics. The fluorescence value of the CDKN2B 3′UTR + NC mimics group was set at 1.0. Three independent experiments were performed with each group. ^*^P<0.05. CDKN2B, cyclin-dependent kinase inhibitor 2B; miR, microRNA; 3′UTR, 3′ untranslated region; NC, negative control; mut, mutated.

To investigate the regulatory mechanism of miR-708 and miR-31 in CRC, the target gene for these miRs was investigated using bioinformatics. The target gene prediction was based on the following principles: i) The candidate gene has binding sites with miRs in the 3′UTR; ii) the candidate gene exerts similar roles to that of the miRs; and iii) the candidate gene is expressed in CRC; CDKN2B was selected as the target gene for further investigation. Fig. 5A and B show the alignment of miR-31 and miR-708 with CDKN2B 3′UTR and mutant 3′UTR. To validate whether the two miRs regulated CDKN2B 3′UTR directly through the binding sites, the wild-type and mutant-type of CDKN2B were constructed and cloned into the downstream of a luciferase reporter gene. The results from the luciferase reporter assay demonstrated that miR-31 and miR-708 reduced the luciferase intensity of wild-type CDKN2B 3′UTR by ~70 and ~50%, respectively, compared with the miR-NC cells. However, mutation in the binding sites of the CDKN2B 3′UTR reduced the ability of these two miRs to inhibit luciferase activity, indicating that they bind directly to the 3′UTR of CDKN2B (Fig. 5C and D).

### miR-708 and miR-31 negatively regulate CDKN2B expression levels

**Figure 6:**
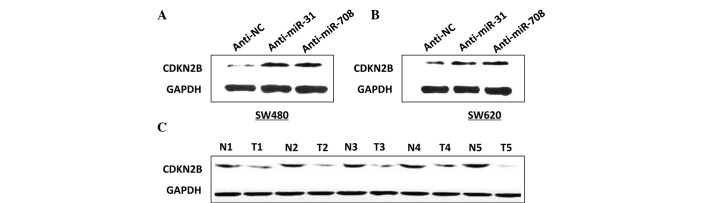
CDKN2B expression was downregulated in colon cancer tissues and negatively regulated by miR-31 and miR-708 in the human cell line cells, SW480 and SW620. (A and B) The expression of CDKN2B was increased in the cells with anti-miR-31 or anti-miR-708 in the SW480 and SW620 cells. (C) Representation of the downregulated CDKN2B levels in the CRC tissues compared with the adjacent healthy tissues. CDKN2B, cyclin-dependent kinase inhibitor 2B; miR, microRNA; CRC, colorectal cancer; NC, negative control; N, adjacent normal tissue; T, tumor tissue.

**Figure 7:**
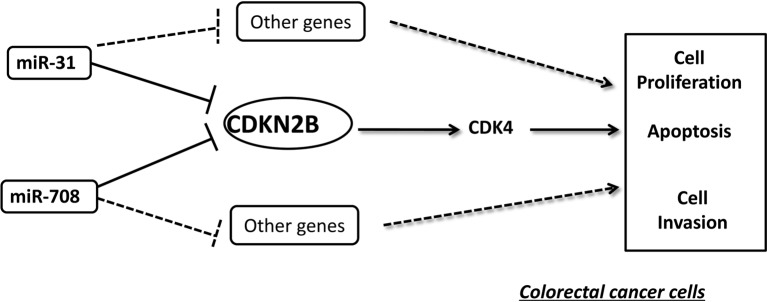
Schematic representation of the hypothetical molecular mechanism of miR-31 and miR-708 that regulates the proliferation, apoptosis and invasion of CRC cells through targeting CDKN2B. Dashed lines represent a predicted result, while solid lines represent the findings of the present study. miR, microRNA; CRC, colorectal cancer; CDKN2B, cyclin-dependent kinase inhibitor 2B.

To determine the effect of the two miRs on CDKN2B expression levels, western blot analysis was conducted. Fig. 6A shows that the expression of CDKN2B protein levels was increased in the cells with anti-miR-31 or anti-miR-708, compared with the anti-NC cells (Fig. 6A and B). In addition, CDKN2B protein levels were investigated in five CRC tissues and the adjacent healthy tissues. Fig. 6C indicates that the CDKN2B levels were lower in the CRC tissue samples, which contrasts with the miR-31 and miR-708 expression levels. Taken together, these results indicate that CDKN2B is negatively regulated by miR-31 and miR-708. This negative regulation is also schematically demonstrated in Fig. 7.

## Discussion

Increasing evidence shows that 50% of miRs are located in the fragile or cancer-gene associated regions of chromosomes (17), indicating that aberrant miR expression is closely associated with cancer development. Therefore, to investigate the roles of miRs in cancer growth and metastasis, it is important to validate the deregulated miRs. Using miR profiling, previous studies have demonstrated that miR-708 and miR-31 are upregulated in CRC (18,19), and the effect of miR-31 in CRC cells has been partially evaluated (20). However, there are few studies investigating the roles of miR-708 in CRC. Using qPCR analysis, the present study identified that miR-708 and miR-31 were overexpressed in CRC tissues, when compared with healthy colon tissue samples, which was consistent with prior data (18).

Cell growth and invasion are important characteristics for cancer cells. In the present study, cell growth was initially investigated using the MTT assay and apoptosis analysis. The MTT results indicated that inhibition of miR-31 and miR-708 suppressed the cell viability. The Annexin V assay, which was conducted for apoptosis analysis, found that inhibition of miR-31 and miR-708 led to an induction of cell apoptosis. In addition, a Transwell chamber assay was performed for the invasion assay, indicating that anti-miR-31 and anti-miR-708 resulted in the reduction of cell invasion. Accordingly, the effect of miR-31 and miR-708 in CRC cells has been observed in other types of cancer. For example, miR-31 is upregulated in esophageal cancer and promotes cancer development via suppression of its target gene, PPP2R2A (21). These data indicate that miR-31 and miR-708 may be significant in tumor growth and metastasis, however, this requires further investigation *in vivo.*

The results of the functional investigation of the two miRs in the present study indicate that miR-31 and miR-708 exert a similar effect on CRC cell growth and invasion. To validate whether these two miRs cooperatively regulate the behaviors of CRC cells, it was considered crucial to determine the common target gene for the two miRs. Bioinformatics was used for target gene prediction. CDKN2B was finally selected from the various candidate genes. The luciferase reporter assay, a direct method for target gene validation, subsequently identified that the two miRs reduced the CDKN2B 3′UTR intensity, while neither miR-31 nor miR-708 had an effect on CDKN2B 3′UTR that contained the mutant binding sites. The western blot assay showed that miR-31 and miR-708 reduced CDKN2B protein levels. In addition, there were low CDKN2B protein levels in the CRC tissues that had high miR-31 and miR-708 expression levels. These results identified that CDKN2B was a direct target gene for miR-31 and miR-708, thus, indicating that one gene may be regulated by multiple miRs. Certain studies also indicate that multiple miRs regulate the same gene expression through binding to various sites in the 3′UTR of a target gene. For example, miR-30d, miR-181a and miR-199a-5p cooperatively target GRP78 in prostate, colon and bladder tumors and cancer cell lines (22).

Furthermore, one gene may be modulated by multiple miRs, however, one miR is also able to regulate numerous genes. The present study showed that miR-31 and miR-708 target CDKN2B. A previous study indicated that miR-31 is important in vascular smooth muscle cell growth via the suppression of LATS2 (23). miR-708 promotes bladder cancer cell growth and inhibits cell apoptosis by targeting caspase-2 (24). However, the present study indicated that CDKN2B may partially mediate the functions of miR-31 and miR-708 in CRC.

In the present study, CDKN2B expression was found to be downregulated in CRC tissues, which is consistent with the transcriptome profile of colorectal adenomas (25). Previous studies have identified that there is a high frequency of methylation in the promoter of CDKN2B, leading to the downregulation of CDKN2B in colon cancer (26,27). Therefore, it was hypothesized that the low protein levels of CDKN2B in the present study may also be due to hypermethylation, however, this requires further investigation. Certain studies have indicated that CDKN2B binds to cyclin-dependent kinase complexes (CDKCs; CD/CDK4 or CD/CDK6) during the cell cycle transition, in particular at G1/S, resulting in cell cycle arrest at the G1/S transition and cell proliferation inhibition (28,29). In addition, endoplasmic reticulum protein 29 suppresses breast cancer cell invasion by upregulating numerous genes, including CDKN2B, indicating that CDKN2B is involved in cell invasion (30). The roles of CDKN2B oppose those of miR-31 and miR-708, which further indicates that these two miRs may regulate CRC cell growth and invasion through the suppression of CDKN2B, however, this requires further investigation.

In conclusion, it was demonstrated that miR-31 and miR-708 were upregulated in CRC, and inhibition of the two miRs induced the reduction of cell growth and invasion; the miRs function as oncogenes. In addition, a direct target gene, CDKN2B, was identified for miR-31 and miR-708 and these data indicate that the two miRs may act via the suppression of CDKN2B (Fig. 7). Therefore, miR-31 and miR-708 may be involved in a therapeutic strategy for patients as novel biomarkers for the diagnosis and prognosis of CRC.

## References

[1] Zhang Y, He X, Liu Y (2012). microRNA-320a inhibits tumor invasion by targeting neuropilin 1 and is associated with liver metastasis in colorectal cancer. Oncol Rep.

[2] Baraniskin A, Birkenkamp-Demtroder K, Maghnouj A (2012). MiR-30a-5p suppresses tumor growth in colon carcinoma by targeting DTL. Carcinogenesis.

[3] Braconi C, Kogure T, Valeri N (2011). microRNA-29 can regulate expression of the long non-coding RNA gene MEG3 in hepatocellular cancer. Oncogene.

[4] Chang Y, Yan W, He X (2012). miR-375 inhibits autophagy and reduces viability of hepatocellular carcinoma cells under hypoxic conditions. Gastroenterology.

[5] Bartel DP (2004). MicroRNAs: genomics, biogenesis, mechanism, and function. Cell.

[6] Zeng Y, Yi R, Cullen BR (2003). MicroRNAs and small interfering RNAs can inhibit mRNA expression by similar mechanisms. Proc Natl Acad Sci USA.

[7] Zhang LY, Liu M, Li X, Tang H (2012). miR-490-3p modulates cell growth and epithelial to mesenchymal transition of hepatocellular carcinoma cells by targeting endoplasmic reticulum-Golgi intermediate compartment protein 3 (ERGIC3). J Biol Chem.

[8] Brennecke J, Hipfner DR, Stark A, Russell RB, Cohen SM (2003). bantam encodes a developmentally regulated microRNA that controls cell proliferation and regulates the proapoptotic gene hid in Drosophila. Cell.

[9] Xu P, Vernooy SY, Guo M, Hay BA (2003). The Drosophila microRNA Mir-14 suppresses cell death and is required for normal fat metabolism. Curr Biol.

[10] Ambros V (2004). The functions of animal microRNAs. Nature.

[11] Chen CZ, Li L, Lodish HF, Bartel DP (2004). MicroRNAs modulate hematopoietic lineage differentiation. Science.

[12] Bai S, Nasser MW, Wang B (2009). MicroRNA-122 inhibits tumorigenic properties of hepatocellular carcinoma cells and sensitizes these cells to sorafenib. J Biol Chem.

[13] Tsai WC, Hsu PW, Lai TC (2009). MicroRNA-122, a tumor suppressor microRNA that regulates intrahepatic metastasis of hepatocellular carcinoma. Hepatology.

[14] Nie J, Liu L, Zheng W (2012). microRNA-365, down-regulated in colon cancer, inhibits cell cycle progression and promotes apoptosis of colon cancer cells by probably targeting Cyclin D1 and Bcl-2. Carcinogenesis.

[15] Li Q, Zou C, Han Z (2013). MicroRNA-25 functions as a potential tumor suppressor in colon cancer by targeting Smad7. Cancer Lett.

[16] Tang F, Zhang R, He Y (2012). MicroRNA-125b Induces metastasis by targeting STARD13 in MCF-7 and MDA-MB-231 breast cancer cells. PloS One.

[17] Calin GA, Sevignani C, Dumitru CD (2004). Human microRNA genes are frequently located at fragile sites and genomic regions involved in cancers. Proc Natl Acad Sci USA.

[18] Piepoli A, Tavano F, Copetti M (2012). Mirna expression profiles identify drivers in colorectal and pancreatic cancers. PLoS One.

[19] Necela BM, Carr JM, Asmann YW, Thompson EA (2011). Differential expression of microRNAs in tumors from chronically inflamed or genetic (APC(Min/+)) models of colon cancer. PLoS One.

[20] Cekaite L, Rantala JK, Bruun J (2012). MiR-9, -31, and -182 deregulation promote proliferation and tumor cell survival in colon cancer. Neoplasia.

[21] Alder H, Taccioli C, Chen H (2012). Dysregulation of miR-31 and miR-21 induced by zinc deficiency promotes esophageal cancer. Carcinogenesis.

[22] Su SF, Chang YW, Andreu-Vieyra C (2012). miR-30d, miR-181a and miR-199a-5p cooperatively suppress the endoplasmic reticulum chaperone and signaling regulator GRP78 in cancer. Oncogene.

[23] Liu X, Cheng Y, Chen X (2011). MicroRNA-31 regulated by the extracellular regulated kinase is involved in vascular smooth muscle cell growth via large tumor suppressor homolog 2. J Biol Chem.

[24] Song T, Zhang X, Zhang L (2013). miR-708 promotes the development of bladder carcinoma via direct repression of Caspase-2. J Cancer Res Clin Oncol.

[25] Sabates-Bellver J, Van der Flier LG, de Palo M (2007). Transcriptome profile of human colorectal adenomas. Mol Cancer Res.

[26] Nieminen TT, Shoman S, Eissa S, Peltomäki P, Abdel-Rahman WM (2012). Distinct genetic and epigenetic signatures of colorectal cancers according to ethnic origin. Cancer Epidemiol Biomarkers Prev.

[27] Gonzalez-Zulueta M, Bender CM, Yang AS (1995). Methylation of the 5′ CpG island of the p16/CDKN2 tumor suppressor gene in normal and transformed human tissues correlates with gene silencing. Cancer Res.

[28] Shi T, Mazumdar T, Devecchio J (2010). cDNA microarray gene expression profiling of hedgehog signaling pathway inhibition in human colon cancer cells. PLoS One.

[29] Choi S, Kim TW, Singh SV (2009). Ginsenoside Rh2-mediated G1 phase cell cycle arrest in human breast cancer cells is caused by p15 Ink4B and p27 Kip1-dependent inhibition of cyclin-dependent kinases. Pharm Res.

[30] Bambang IF, Xu S, Zhou J (2009). Overexpression of endoplasmic reticulum protein 29 regulates mesenchymal-epithelial transition and suppresses xenograft tumor growth of invasive breast cancer cells. Lab Invest.

